# Relationship Types and Care Received: Differences by Dementia Status

**DOI:** 10.1093/geroni/igaf122.1166

**Published:** 2025-12-31

**Authors:** Sarah Patterson

**Affiliations:** University of Michigan, Ann Arbor, Michigan, United States

## Abstract

Families and unpaid caregivers continue to provide the majority of assistance to older Americans, including to older adults living with dementia. However, diversity in the family structures of older adults may alter the help they receive and from whom. Using the 2022 National Health and Aging Trends (NHATS) study, I find older adults with and without dementia are equally likely to report having any children or siblings. Although older adults with dementia are more likely to be unpartnered, rates of care by a partner are the same once accounting for partnership status. Older adults with dementia are significantly more likely than those without dementia to receive care from children and grandchildren, but rates of care received are similar for siblings in both groups. These findings have implications for understanding the broader care networks of older adults beyond the traditional focus on a spouse and adult children.

